# An Alternative Route to Obtain Carbon Quantum Dots from Photoluminescent Materials in Peat

**DOI:** 10.3390/ma11091492

**Published:** 2018-08-21

**Authors:** Rafael Souza da Costa, Wiliam Ferreira da Cunha, Nizamara Simenremis Pereira, Artemis Marti Ceschin

**Affiliations:** 1 Electrical Engineering Department, Universidade de Brasília, Brasília DF 70919-970, Brazil; faelsc@gmail.com; 2 Instituto de Física, Universidade de Brasília, Darcy Ribeiro Campus, Brasília DF 70919-970, Brazil; wiliamcunha@gmail.com; 3 Instituto Federal de Brasília, Campus Gama, Setor Central do Gama, Brasília DF 72405-025, Brazil; nizamara.pereira@ifb.edu.br

**Keywords:** peat talc, carbon dots, photoluminescence, humic substances, organic electronics

## Abstract

Peat, an organic compound easily found in the soil (easy to acquire), has more than 50% elemental carbon in its composition and can be used as raw material to produce carbon quantum dots (CQDs, C-dots, Carbon Dots). In this work we describe two simple and low-cost routes for the acquisition of these photoluminescent materials based on peat. The final products were characterized by Fourier transform infrared spectroscopy (FTIR), absorption (UV-Vis) and emission (PL) spectra and high-resolution transmission electron microscopy (HRTEM). The produced CQDs have an average size of 3.5 nm and exhibit coloration between blue and green. In addition, it is possible to produce photoluminescence by means of the aromatic compounds also present in the composition of the peat, in turn exhibiting an intense green coloration. The results indicate great versatility of peat for the production of photoluminescent materials.

## 1. Introduction

Quantum dots (QDs) are some of the most prominent materials in nanotechnology. They consist of nanometer-sized semiconducting species (usually ranging from 2 to 10 nm) whose properties might significantly vary from both the bulk and the molecular version of the material [1,2,3,4,5,6,7]. The discovery of the inorganic version of quantum dots dates back to 1982, whereas their carbon counterparts, named carbon quantum dots (CQDs), were discovered in 2004.

**Figure 1:**
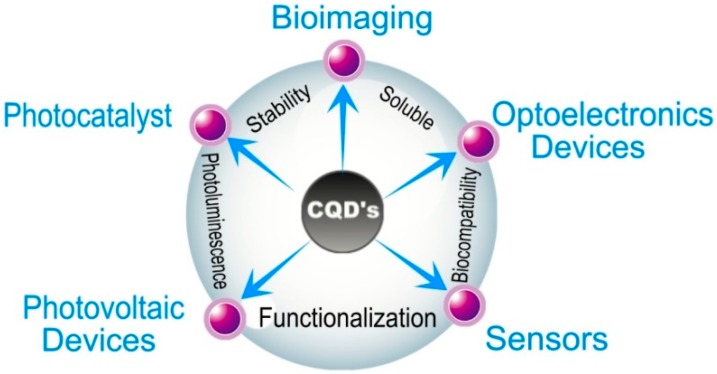
Main properties and applications of CQDs in the Organic Electronics field. The figure was drawn based on the model stated on the Reference [7].

Quantum dots in general have gained attention from the scientific community for the high amount of potential applications, such as catalysis, sensing and bioimaging, the development of optoelectronic devices, photovoltaic cells, field-effect transistors, photodetectors, light-emitting diodes, among other applications of great relevance to Electronics in general [3,4,8,9,10,11]. However, the synthesis and functionalization of inorganic QDs might involve the use of heavy metals such as cadmium, and the most efficient solar cells known to date, produced with quantum dots and perovskite, contain lead. Thus, there is a considerable level of toxicity associated with these nanomaterials. Carbon quantum dots, on the other hand are non-toxic, and have a variety of properties that make them unique, as well as being the likely substitutes of inorganic nanocrystals. Some CQD characteristics and properties, as well as the potential applications in electronics [4,5,6,7,8,12,13,14,15,16,17] are shown in Figure 1.

In order for a process to take its place, CQDs obtained from different sources ought to be completely characterized and understood. It is only by gaining this kind of knowledge that CQD-based devices can achieve and perhaps surpass the efficiency of the conventional ones. Importantly, it would be highly desirable to investigate CQDs that originate from sustainable sources, as the impact of the electronics industry on the environment is one of the key advantages of CQDs over conventional QDs. If this is the case, one needs first to characterize the very source of the production of CQDs. In the present study, the stock material for the production of carbon quantum dots will be an “*in natura*” substance known as peat, or substrate for plants. This substance has a high amount of carbon present in its chemical structure [18].

Peat is a formidable material for research, formed by three distinct fractions: humic acid (soluble in alkaline media), fulvic acid (soluble in both acid and alkaline media) and humina (insoluble in both acid and alkaline media), which in the scientific world are commonly referred to as humic substances (HSs) [18,19,20,21]. Each of these fractions has the potential to provide many different applications for the material. It is important to remember that there is a large debate regarding the chemical structure of each of these parts that constitute peat, and this fact is enough to turn peat into a material of interest for academic research. Despite being a long-established organic material for agricultural activity, this is far from the only possibility of application for peat, as studies in the engineering field look for new utilities for this material [21,22,23]. Peat’s main contribution to recent studies is the quality of the carbon present in its composition. Besides this characteristic there is the crucial fact that it is an easily acquired material, with low cost and low toxicity.

In recent studies, there have been reports of the use of humic substances in the production of ammonia and humidity sensors [21,22]. Y. Dong et al. [23] proposed the production of CQDs from the soluble phases of peat (humic acid and fulvic acid), which are also abundant sources of carbon.

The present work recommences studies undertaken in 2012 by our own research group, which utilized humina (one of the fractions of peat) as the raw material for the manufacture of an ammonia sensor [20,21,22], opening space for a new field of study utilizing the same material for the production of carbon quantum dots. Y. Dong et al. [23] used humic substances (HSs), which are peat byproducts, for the production of carbon quantum dots. Their CQD preparation methodology, however, was quite different from the one proposed in the present work. One of the crucial divergent points is that Y. Dong et al. utilized independent peat fractions to synthesize CQDs, utilizing each peat soluble fraction separately, humic acid or fulvic acid. Our paper on the use of three fractions that constitute peat allows a greater analysis of carbon atoms in the sample.

In this study, we utilized peat talc [20,21,22] to facilitate two routes of preparation of two different types of materials with photoluminescent properties. Route I is similar to the one proposed by Y. Dong et al., and it does not involve synthesis. Our results indicate that the luminescence of the material obtained from the humic substances (fulvic and humic acid) derives from the aromatic compounds and not from CQDs. The preparation of the photoluminescent materials through Route II provided us with CQDs through the synthesis of peat talc. The preparation of these carbon quantum dots was carried out by two chemical methods: thermal pyrolysis (synthesis) and hydrothermal (extraction). We detail the peat talc’s preparation and its characterization by X-ray Diffraction (XRD), Raman spectroscopy and by Fourier-Transform Infrared Spectroscopy (FTIR). Both the materials containing aromatic and heteroaromatic compounds, as well as those containing CQDs, were characterized by absorption (UV-Vis) and emission (PL) spectra. Only the sample containing CQDs was analyzed with High-Resolution Transmission Electron Microscopy (HRTEM). Although the sample with HSs was analyzed with HRTEM, due to its high concentration of sodium hydroxide, it was not possible to complete the test.

## 2. Experimental Section

### 2.1. Materials and Methods

The “*in natura*” peat sample was donated by Florestal, S.A. enterprise, with headquarters in the cities of Criciúma and Balneário Arroio do Silva, both in the state of Santa Catarina, southern region of Brazil. The peat sample went through a process of removal of part of its humidity in an oven at 80 °C for 24 h. The peat with part of its humidity removed was crushed mechanically with the aid of a mortar and pestle. The obtained material passed through a sieve with pores of 420 µm. The resulting material has the appearance of a very fine powder, which is why we refer to it as peat talc.

This peat talc was characterized by X-ray diffraction in a D8 Focus diffractometer (Bruker, Billerica, MA, USA). The diffractogram was generated utilizing a wavelength of 1.54 Å, a step size of 0.05°, scan speed of 0.5° min^−1^, and a scanning range between 0° and 90°.

The Raman spectroscopy measurements were taken at room temperature, with equipment manufactured by HORIBA Scientific (Kyoto, Japan), model T64.000, linewidth of 514, 532, 633 and 785 nm, with laser power of 15 mW, configured for backscattering. The detection of the signal of spread light was done by a CCD camera, model Synapse. The T64.000 was connected to an Olympus BX 41 microscope (Tokyo, Japan) and a 10× objective lens. The FTIR spectrum was measured in a Spectrum Two spectrometer by PerkinElmer (Waltham, MA, USA), at room temperature, in the range of 4000 to 400 cm^−1^ (medium infrared range).

The absorption spectrum was obtained with an Evolution 300 UV-Vis spectrophotometer by Thermo Fisher Scientific (Waltham, MA, USA), with an initial scanning range of 190 to 800 nm. Photoluminescence was performed in a fluorescence spectrophotometer by PerkinElmer, model L555, with a slit aperture equal to 3, excitation wavelength of 270 nm, and scanning range of 450 to 800 nm.

The samples were analyzed by electron microscopy, under a transmission electron microscope (TEM, Jeol, JEM-2100 (Tokyo, Japan), equipped with energy dispersive spectrometer (EDS) by Thermo Scientific).

### 2.2. Extraction of Fluorescent Humic Substances from Peat (Route I)

Route I provides a material rich in aromatic and heteroaromatic compounds. The employed method is extraction by means of dissolution of peat talc in sodium hydroxide at the concentration of 0.1 mol/L (lower efficiency in the extraction) to 1 mol/L (higher efficiency in the extraction) at the proportion of 1:10.4 g of peat talc solubilized in 40 mL of sodium hydroxide [18]. This mixture was stirred for 4 h until it became homogeneous. Next, this mixture was centrifuged at 5000 rpm for 10 min. The liquid phase was discarded, and the solid phase was washed with deionized water and put in an oven at 80 °C for water elimination. After drying, the solid phase was once again solubilized with magnetic stirring for 30 min, in 40 mL of deionized water at 50 °C, to facilitate the dissolution process. To dissolve the solid part, 30 min of stirring was needed. Finally, the suspension was allowed to rest so that the solid particles would decant and the temperature would drop until reaching room temperature. The supernatant portion was removed and filtered with a vertical membrane syringe filter of 0.45 µm thickness. The filtrate has two fractions of the peat: fulvic acid and humic acid, since humina is not soluble in a liquid base. Due to the liquid having a dark brown color, around 0.02 mL of the filtrate was diluted in 10 mL of deionized water, in order to perform the absorption and emission spectroscopies of the sample in question.

### 2.3. Synthesis of Carbon Quantum Dots (Route II)

Route II provides the carbon quantum dots. Approximately 10 g of the peat talc was calcinated in a muffle with a heating ramp of 10 °C/min and thresholds of 400, 500, 750 and 1000 °C for 2 h. The CQD production process consists of two simple steps: the peat calcination, and the CQD extraction with a sodium hydroxide solution. The peat calcination was conducted at 1000 °C for 2 h. After the cooling of the sample, 1 g of the calcinated peat is solubilized in 100 mL sodium hydroxide 1 mol L^−1^ which remains in constant agitation, and heating of about 50 °C for 2 h. After this interval, the solution is allowed to rest for around 2 h. We observed a phase separation and collected the supernatant only, which should contain the carbon quantum dots. The supernatant is filtered in a 0.45-µm membrane filter.

## 3. Results and Discussion

### 3.1. XRD

**Figure 2:**
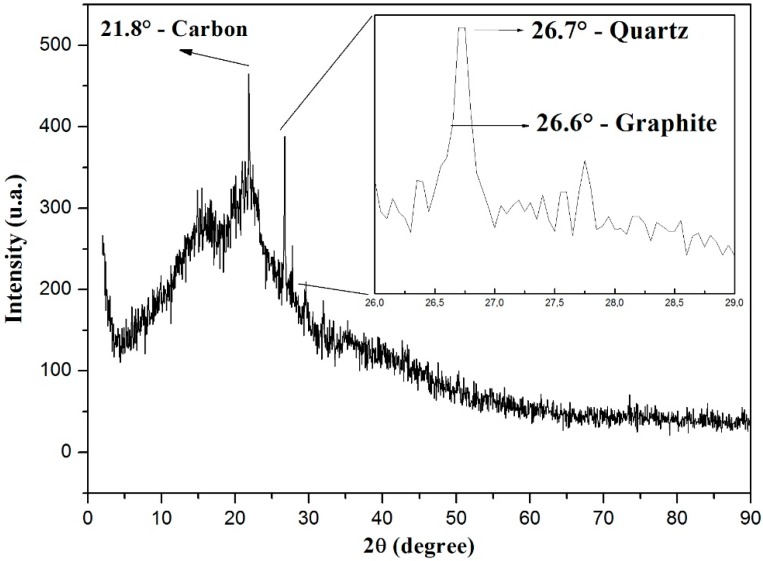
XRD standard shown by the peat sample as amorphous material.

The peat talc presents itself as an amorphous material according to the XRD shown in Figure 2. In the range between 8° and 30°, we observe that there is a broad band related to several compounds containing carbon, and three more pronounced peaks over it. By means of the Diffrac Eva software (Bruker, Billerica, MA, USA), it was possible to identify these three relevant peaks of Figure 2. The peak at 21.8° is related to elemental carbon; the one at 26.6° is related to graphite, according to what was observed in reference [24]; finally, the peak at 26.7° is related to quartz.

The presence of the elemental carbon was expected, due to the peat composition with humic acid, fulvic acid, and humin.

### 3.2. Raman Spectroscopy

**Figure 3:**
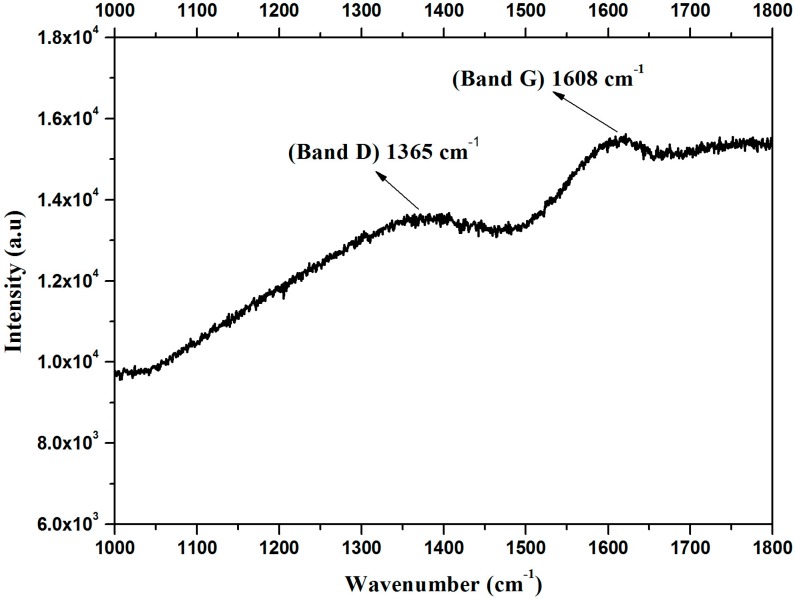
Raman spectrum of the peat talc sample.

The Raman spectrum of the peat sample (Figure 3) shows a characteristic D (disorder) band at 1365 cm^−1^, which is attributed to structural defects (probably originated from amorphous carbon due to the peak’s width) and a G (graphite) band at 1608 cm^−1^, attributed to the first order scattering vibration mode E2g in the graphite sheet [23]. From the Raman spectrum it is possible to state that the peat sample has relatively abundant carbon structures in the form of graphite. The intensity ratio (I_D_/I_G_), which indicates the concentration of defects, is in the order of 0.85. This factor shows that a high concentration of defects is present in the sample. The fact that peak G was found outside its characteristic region, i.e., near 1580 cm^−1^, might be related to the elevated number of hydrogen atoms that are part of peat’s chemical structure, since the presence of hydrogen atoms is known to cause the band G to shift to higher wavelengths [25].

### 3.3. FTIR

**Figure 4:**
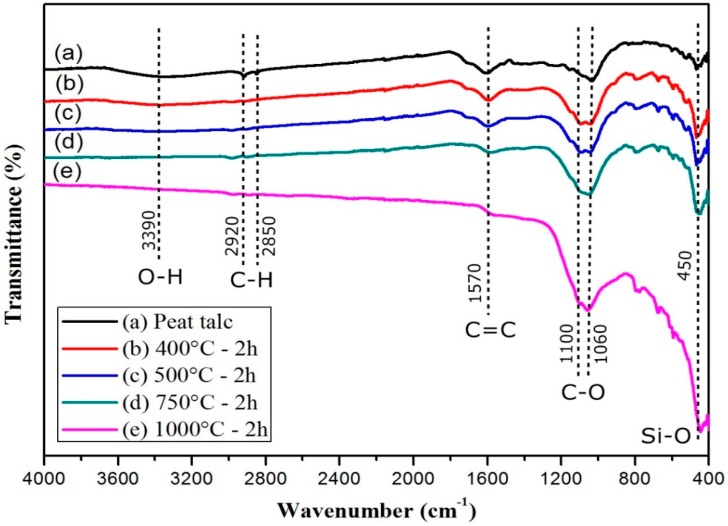
Absorption spectroscopy on the infrared range (FTIR), medium range for the peat talc and portions of the same sample undergoing different calcination temperatures: (**a**) peat “*in natura*”, (**b**) 400 °C, (**c**) 500 °C, (**d**) 750 °C, and (**e**) 1000 °C.

The FTIR absorption spectroscopy results for the peat talc are shown in Figure 4. For the peat talc, seen in the spectra, a few organic groups can be observed. O–H systems of carboxylic acids present axial stretching and show in a wide band in the region of 3600 to 3200 cm^−1^ centered in 3390 cm^−1^; symmetrical and asymmetrical axial stretching of CH_2_ systems is situated in 2920 and 2850 cm^−1^ respectively; C=C aromatic nuclei vibrations are found in the region of 1570 cm^−1^; C–O axial stretching in alcohols and phenols is related to the strong absorption bands in 1100 and 1060 cm^−1^ [18]; and, finally, impurities resulting from vibration modes of silicates are in the band centered at 450 cm^−1^. The result shown in Figure 4a is in agreement with that reported by Morais et al. [26] and Silverstein et al. [27].

Figure 4b–e spectra refer to the calcinated peat at the temperatures of 400, 500, 750, 1000 °C, respectively. It is possible to notice that for temperatures lower than 1000 °C, the aromatic compounds (C=C) are not affected. Note that the peak centered in the region of 1570 cm^−1^ lowers at the same rate that the temperature rises, particularly for the sample subjected to 1000 °C. Taking curve (a) as reference, and the other curves as a comparison, the evidence that most strongly proves the changes occurred in the samples is related to the peaks in the regions of 1100 and 1060 cm^−1^ respectively, which become more evident at the rate at which the temperature rises. This fact might be related to the lower gradual disorder and greater amount of these compounds in the sample as the temperature rises. Furthermore, the broad band centered in 3390 cm^−1^ ceased to exist. The bands in 2920 and 2850 cm^−1^ became almost imperceptible for the four processes of calcination.

**Figure 5:**
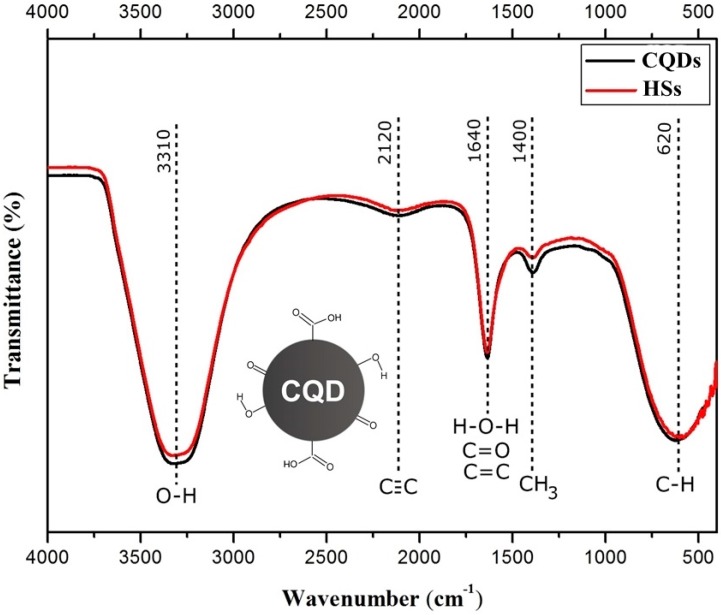
Absorption spectroscopy on the infrared range (FTIR), medium range for solutions containing carbon quantum dots (CQDs) and humic substances (HSs). The inserted figure of a CQD shows the main groups that can be associated with the carbon structure.

The results of the absorption spectroscopy FTIR for the CQDs and for the HSs are shown in Figure 5. The two samples presented very similar results, since there is superposition in several areas of the spectra.

The samples revealed the several main bands. The one between 3300–3500 cm^−1^, present in both spectra, is usually attributed to OH stretching and H bonds in COOH groups, but can also be attributed to other functional groups such as alcohols, phenols, and even water. The C≡C bond’s weak stretching can be found in the broad absorption centered at 2120 cm^−1^. The band centered at 1640 cm^−1^ covers a region that goes from 1660 to 1600 cm^−1^ and can be formed by the combination of many different absorption phenomena due to other groups present in the sample, in this case causing a superposition of groups. In this way, the peak centered at 1640 cm^−1^ can be attributed to the C=C vibrations of aromatic compounds. However, other groups contributed to the absorption in this region, including C=O of amides groups (called imide bands), non-aromatic double bonds, hydrogen bonds of the C=O group of ketones and quinones, and the symmetrical stretching of the COO^−^ group. The deformation vibrations of the water molecule also occur in this region, centered at 1640 cm^−1^ and will contribute to this absorption if the sample is not completely dry. The absorption at 1400 cm^−1^ can be attributed to the symmetrical deformations of CH_3_. The angular deformations outside of the C–H aromatic rings are responsible for the absorption at 620 cm^−1^.

Concerning the band centered at 1640 cm^−1^ for the HSs produced through Route I, we believe that this absorption is attributed to the C=C vibrations of aromatic compounds. For the CQDs produced through Route II, we believe that this same absorption band represents double bonds of non-aromatic compounds with C=O bonds.

Since the results presented show curves that overlap one another, it is not possible to associate the generated curves with the presence or absence of carbon quantum dots; nonetheless, for the two samples, the presence of organic groups that have carbon in their composition is evident.

In the face of the presented results, and observing the results found by other studies on CQD, such as Jun Ke et al. [28], we believe that the CQDs present a natural functionality in the carbon atom’s surface, which is probably inserted by the synthesis utilized in its fabrication. Once the organic groups present in this layer are precisely characterized, it is possible to insert new groups to alter the photoluminescence of these CQDs to other regions of interest in the light spectrum. Therefore, we can think of a carbon nanoparticle with a structure surrounded by several organic groups similar to CQD inserted in Figure 5.

### 3.4. Absorption Spectroscopy (UV-Vis) and Photoluminescence Spectroscopy (PL)

**Figure 6:**
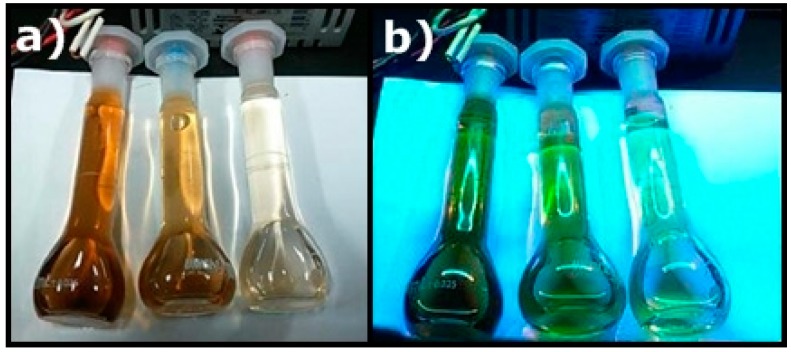
Humic substances produced by route I being stimulated by white light and ultraviolet light. (**a**) HSs under white light. (**b**) HSs under ultraviolet light with wavelength of 254 nm.

The samples produced through Routes I and II were previously studied with the incidence of an ultraviolet light with wavelength of 254 nm. Figure 6 shows three volumetric flasks containing three solutions with different concentrations of humic substances (HSs). In Figure 6a, the samples were subjected to the incidence of a white light. We noticed a brown color, characteristic of the solution with humic substances. We observe that the higher the HSs concentration, the more intense the brown color. In Figure 6b, the same solutions were subjected to an ultraviolet light, and we noted a change in coloring to green. Moreover, the higher the concentration of HSs, the higher the intensity of green. Thus, we conclude that the intensity is directly related to the concentration of the solution containing humic substances.

**Figure 7:**
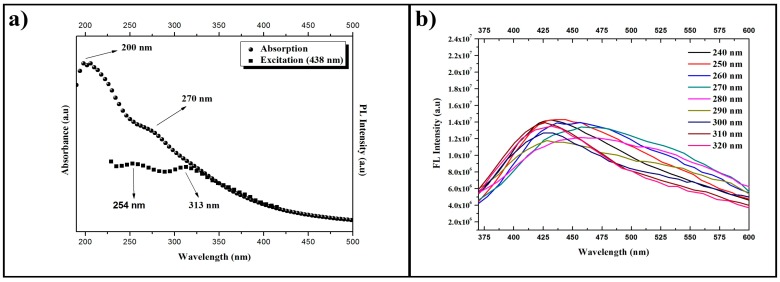
Absorption, excitation, and emission spectra for HSs produced by Route I. (**a**) Absorption curve (spheres) and excitation curve (squares). (**b**) Emission curves (continuous lines) for different excitation wavelengths (increments of 10 nm).

**Figure 8:**
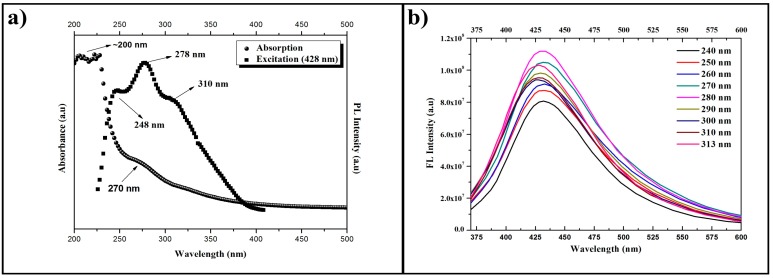
Absorption, excitation, and emission spectra for CQDs produced by Route II. (**a**) Absorption curve (spheres) and excitation curve (squares). (**b**) Emission curves (continuous lines) for different excitation wavelengths (increments of 10 nm).

The absorption, excitation, and emission spectra of the solution produced by Route I (HSs) are presented in Figure 7, whereas the corresponding spectra of the solution produced by Route II (CQDs) are presented in Figure 8. It is possible to observe that the absorption spectra of Figure 7a and Figure 8a have similar behavior, since there is an optical absorption that typically occurs in the ultraviolet range with a tail that elongates to the visible range in both cases. The peaks at 200 nm are attributed to the electronic transitions of the π-π* type in C=C bonds. Meanwhile the peaks at 270 nm are attributed to the n-π* electronic transitions of C=O bonds. These absorption spectra support the results presented by the FTIR band centered at 1640 cm^−1^ as shown in Figure 5, which are also related to C=C and C=O bonds. It is possible to confirm the presence of the two compounds in both samples, although it is not possible to assess the specific proportion through this approach.

The excitation spectra were generated based on the results of the emission spectra. For the HSs, the excitation wavelength utilized was 438 nm, since the highest emission is found on this region. As a result, we obtained two peaks with good absorptions at 254 and 313 nm for the excited state. For the CQDs, the wavelength utilized was 428 nm, since the highest emission occurs in the region next to this wavelength. The obtained results show three peaks with good absorption at 258, 278, and 310 nm for the excited state. The results for the HSs emission curves, with excitations changing at 10 nm intervals, were added in Figure 7b. One can see that, for each increment, the emission wavelength shifts to regions of longer wavelengths, considering excitations of 240 to 280 nm. The emission curves caused by the excitations of 290 to 320 nm have a maximum emission wavelength of 426 nm.

The CQDs presented an emission intensity superior to the one of the HSs samples. The results of the CQDs emission curves, with excitations changing at 10 nm intervals, are presented in Figure 8b. The excitations from 240 to 280 nm produced emissions with wavelengths in the region of 431 nm, and for excitations from 290 to 313 nm, the emission response was shifted to a region of shorter wavelength, in the order of 427 nm, in other words, of higher energy. The main wavelength of maximum emission is that of around 428 nm, which was produced by an excitation of 280 nm. The greater uniformity in size of the nanoparticles in solution is possibly the cause of this strong emission at 431 nm.

**Figure 9:**
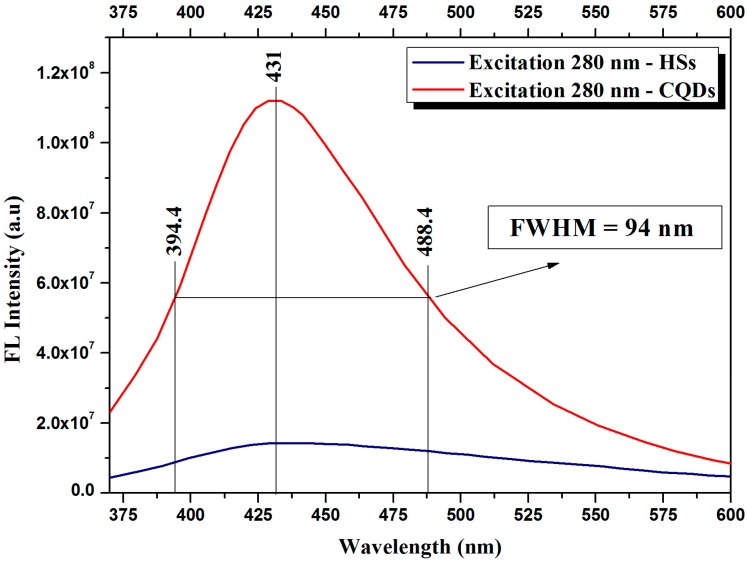
Comparison between the maximum emissions of the samples containing HSs (blue curve) and CQDs (red curve), and width at half maximum of the CQDs’ curve.

In Figure 9 we can better observe the difference between the HSs and CQD absorption spectra. The maximum emission at 431 nm was observed for the two samples. The full width at half maximum (FWHM) of the CQD emission curve is 94 nm.

Both samples present excellent photostability. After an 8 h period of radiation, no loss of fluorescence was noticed. Actually, even after storing the samples for a year (at room temperature and protected from light), the same fluorescence was observed.

### 3.5. High-Resolution Transmission Electron Microscopy

This section deals with information relevant to the prior characterization of CQDs, and the main results obtained with the high-resolution transmission electron microscopy. The previous characterization was carried out by following two procedures. The first one was conducted by the incidence of a laser with wavelength on the region of 640–660 nm (red color) and power of 1 mW. It revealed information concerning the CQDs’ dispersity. The second consisted of submitting the samples to ultraviolet light and reveals CQDs’ fluorescence.

**Figure 10:**
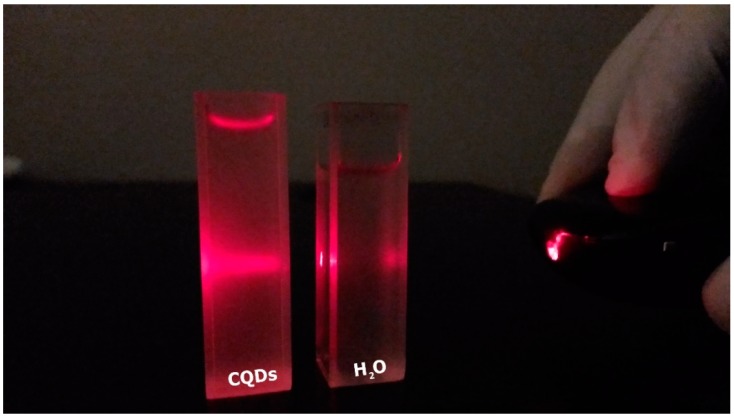
Tyndall effect in a colloid containing carbon nanoparticles.

In order to directly observe the CQDs’ dispersity, derived from peat talc calcinated at 1000 °C, we focused a red laser on the aqueous solution, as shown in Figure 10. As a result, the Tyndall effect appeared on the left-hand cuvette (solution containing CQDs) and was not observed on the right-hand cuvette (plain water). This fact proves that the liquid present in the cuvette containing the CQDs is not a solution, but a colloid, where the nanoparticles are dispersed.

**Figure 11:**
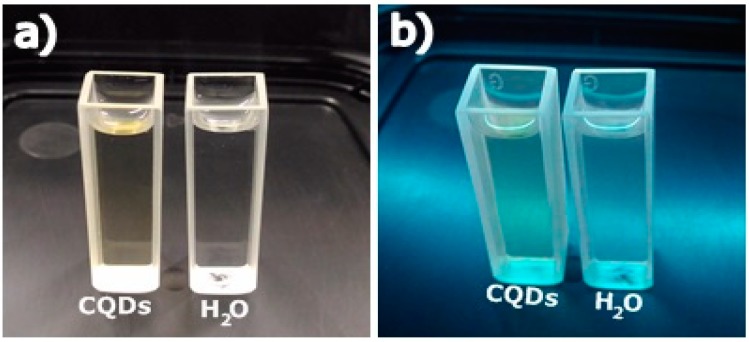
Previous characterization of CQDs. (**a**) Effect of white light on CQDs. (**b**) Effect of ultraviolet light on CQDs (254 nm).

Figure 11a,b show, respectively, the effects of visible and ultraviolet lights on CQDs. It can be observed from Figure 11a that the cuvette containing the CQDs presents a slightly yellowish color, while the other, containing water, is colorless. In Figure 11b, it is possible to observe that the cuvette containing the CQDs presents a slightly blueish color, while the cuvette with plain water does not go through any perceptible change under ultraviolet light. The produced CQDs present typical absorption in the ultraviolet region, and emission in the region of 431 nm, which corresponds to the blue color in the visible light spectrum.

**Figure 12:**
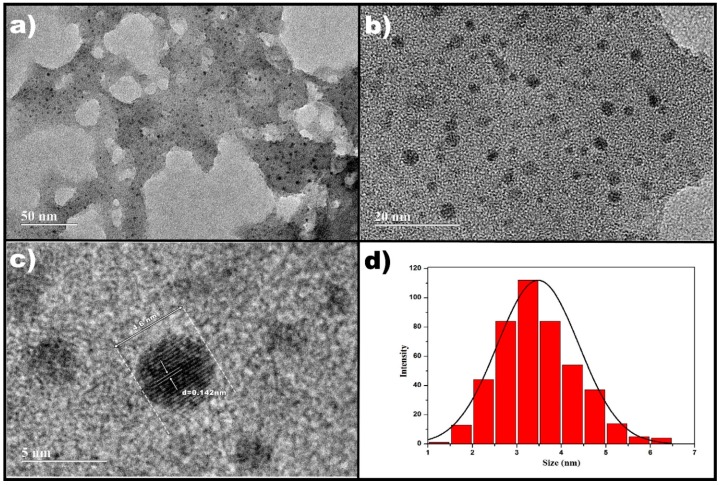
High-resolution transmission electron microscopy. (**a**) Carbon nanoparticles at the scale of 50 nm. (**b**) Carbon nanoparticles at the scale of 20 nm. (**c**) Carbon nanoparticles with approximate diameter of 4.6 nm and highlight of the interatomic distance of 0.142 nm on a 5 nm scale. (**d**) Distribution of the average sizes of CQDs, with average size of 3.5 nm.

Figure 12 shows the results obtained with the high-resolution transmission electron microscopy, and the distribution of the carbon nanoparticles’ average size. Figure 12a,b show the carbon nanoparticles at the scales of 50 and 20 nm, dados corrigidos. respectively. It was possible to observe that the nanoparticles presented an almost circular shape. Figure 12c shows one carbon nanoparticle at the scale of 5 nm. According to the analysis by means of *ImageJ* software (National Institutes of Health, Bethesda, MD, USA), the nanoparticle has an average diameter of approximately 4.6 nm. The lattice spacing is 0.142 nm, which can be attributed to the carbon nanoparticles, since this is the same distance between the carbon atoms in graphite or graphene, considering a small margin of error due to image quality. Because the XRD presented traces of graphite in the peat’s composition, we believe that the nanoparticles are made of carbon atoms that present themselves in the structural form known as graphite. Figure 12d shows the average distribution curve of the carbon nanoparticles that vary from 1 to 8 nm, a fact that proves the nanoparticle’s size non-uniformity. In total, 453 nanoparticles were counted and measured. The statistical analysis by means of the *ImageJ* software determined that these nanoparticles presented an average size of 3.5 nm, which can be determined with the aid of a Gaussian curve.

## 4. Conclusions

The results presented here prove that peat has great potential for the development of new materials for use in organic devices, mainly due to its photoluminescent properties. The characterization by means of X-ray diffraction, and infrared absorption spectrophotometry, demonstrates and proves the presence of carbon structures in the peat sample utilized. Both the XRD and the Raman spectroscopy indicate the presence of graphite structures in the sample of peat, a strong indicator that carbon dots can be found both in the solution containing the CQDs, as well as in the solution containing the HSs. The main characteristic of carbon quantum dots produced from peat is its photoluminescence, which has the potential for use in optoelectronic devices in Organic Electronics, or even in sensors for detecting toxic agricultural inputs in the water of rivers and lakes.

A similar methodology to the one used in the manufacture of the ammonia sensor was utilized in our first attempt to acquire carbon quantum dots. However, instead of the solid portion, the focus now is the liquid part of peat (fulvic and humic acids) that was separated by means of extraction with sodium hydroxide. The result of this extraction generated a dark liquid that, when diluted in water and submitted through ultraviolet light, emits an intense green fluorescence.

By means of peat’s FTIR (peat talc), we noted a very strong influence of aromatic and heteroaromatic groups in the region of 1600 cm^−1^, which was possibly influencing this intense green coloration reported. In this way, the peat talc was subjected to different calcination processes, with the goal of breaking these aromatic and heteroaromatic compounds. We observed a significant change on the region that indicates the presence of these compounds when the sample was subjected to a temperature of 1000 °C. The new extraction made with the peat calcinated at 1000 °C presented now a lighter colored liquid, as we reported, which is similar to that found in the literature for carbon quantum dots.

In this work we presented two routes for the fabrication of photoluminescent materials: Route I, in which we highlight the presence of aromatic and heteroaromatic groups in abundance, and Route II, in which these aromatic compounds are almost eliminated from the sample. In Route II it was possible to identify the carbon nanoparticles in the form of a colloid.

The results with HRTEM indicate the presence of carbon nanoparticles, and the results of the emission and absorption spectra indicate behaviors similar to those found in other studies that focus on the synthesis or production of carbon quantum dots. Having performed the characterization, the next step is to functionalize CQDs so that they will emit fluorescence at other wavelengths, in order to increase their versatility.
